# Pharmacological Manipulation of K_*v*_7 Channels as a New Therapeutic Tool for Multiple Brain Disorders

**DOI:** 10.3389/fphys.2020.00688

**Published:** 2020-06-19

**Authors:** Fabio A. Vigil, Chase M. Carver, Mark S. Shapiro

**Affiliations:** Department of Cellular and Integrative Physiology, University of Texas Health San Antonio, San Antonio, TX, United States

**Keywords:** K_*v*_7, potassium channels, stroke, traumatic brain injury, drug addiction, anxiety, bipolar disorder

## Abstract

K_*v*_7 (“M-type,” KCNQ) K^+^ currents, play dominant roles in controlling neuronal excitability. They act as a “brake” against hyperexcitable states in the central and peripheral nervous systems. Pharmacological augmentation of M current has been developed for controlling epileptic seizures, although current pharmacological tools are uneven in practical usefulness. Lately, however, M-current “opener” compounds have been suggested to be efficacious in preventing brain damage after multiple types of insults/diseases, such as stroke, traumatic brain injury, drug addiction and mood disorders. In this review, we will discuss what is known to date on these efforts and identify gaps in our knowledge regarding the link between M current and therapeutic potential for these disorders. We will outline the preclinical experiments that are yet to be performed to demonstrate the likelihood of success of this approach in human trials. Finally, we also address multiple pharmacological tools available to manipulate different K_*v*_7 subunits and the relevant evidence for translational application in the clinical use for disorders of the central nervous system and multiple types of brain insults. We feel there to be great potential for manipulation of K_*v*_7 channels as a novel therapeutic mode of intervention in the clinic, and that the paucity of existing therapies obligates us to perform further research, so that patients can soon benefit from such therapeutic approaches.

## Introduction

K_*v*_7 channels, also known as M-type, or KCNQ channels, are low-threshold voltage gated K^+^ channels first described almost 40 years ago as underlying the cholinergic slow excitatory post-synaptic potential in sympathetic neurons (Brown and Adams, 1980; Constanti and Brown, 1981). K_*v*_7 channels can be composed of homo- or heterotetrameric assembly of K_*v*_7.1-K_*v*_7.5 subunits; however, only K_*v*_7.2-5 are expressed in the nervous system (Jentsch, 2000). In a wide variety of central and peripheral neurons, M-channels play a significant role in controlling active and passive discharge properties, including action potential threshold, resting membrane potential, spike afterhyperpolarization (AHPs), and shunting conductance (Jones et al., 1995; Yue and Yaari, 2004; Peters et al., 2005; Shah et al., 2008). Consistent with that role, channels composed of K_*v*_7.2 and 7.3 in varying composition are mainly localized in brain to the axon initial segment (Cooper et al., 2001; Pan et al., 2006; Rasmussen et al., 2007), adjacent to the Na_*V*_ channels that generate action potentials. Since M channels deactivate slowly, they contribute to AHP currents (Tzingounis and Nicoll, 2008). Excessive K_*v*_7-channel suppression or channel dysfunction often leads to seizures or other epileptic syndromes (Biervert et al., 1998; Singh et al., 1998; Ambrosino et al., 2015; Miceli et al., 2015; Greene and Hoshi, 2017), leading to the idea that proper M-channel function acts as a “brake” to prevent excess hyperexcitability (Maljevic et al., 2008; Soldovieri et al., 2011), by accumulated M-current activation increasing the threshold for firing (Peters et al., 2005; Tzingounis and Nicoll, 2008) and increasing the interspike interval (Lawrence et al., 2006).

M current is so named for its discovery as a K^+^ conductance suppressed by stimulation of muscarinic acetylcholine receptors in sympathetic ganglia neurons (Brown et al., 1995). In those cells, the action is via G_*q/*__11_-mediated activation of phospholipase C, which hydrolyzes phosphatidylinositol-4,5-bisphospate (PIP_2_), reducing its abundance in the membrane (Haley et al., 1998; Suh and Hille, 2005), and by activation of protein kinase C (Hoshi et al., 2003). Since PIP_2_ binding is required for M-channel opening (Zhang et al., 2003; Li et al., 2005; Suh et al., 2006; Sun and MacKinnon, 2020), its depletion reduces M-current amplitudes in a voltage-independent manner (Shapiro et al., 2000; Nakajo and Kubo, 2005; Choveau et al., 2018). However, in those same neurons, other G_*q/*__11_-coupled receptors suppress M current via release of Ca^2+^ from IP_3_-gated stores, loading of Ca^2+^ into calmodulin (Gamper and Shapiro, 2003; Winks et al., 2005; Zaika et al., 2007) and changes in configuration of CaM molecules bound to the proximal carboxy terminus of K_*v*_7.1-7.5 subunits (Yus-Najera et al., 2002; Haitin and Attali, 2008) in varying configurations (Haitin and Attali, 2008; Kosenko and Hoshi, 2013; Strulovich et al., 2016; Sun and MacKinnon, 2017; Chang et al., 2018; Archer et al., 2019).

Pharmacological manipulation of M current has been studied extensively as a therapeutic option for epilepsy (Kapetanovic et al., 1995; Rostock et al., 1996; Armand et al., 1999; Miceli et al., 2008; Amabile and Vasudevan, 2013; Splinter, 2013) and for analgesia (Blackburn-Munro and Jensen, 2003; Munro and Dalby-Brown, 2007; Szelenyi, 2013; Hayashi et al., 2014; Abd-Elsayed et al., 2015; Zheng et al., 2015; Busserolles et al., 2016; Wang and Li, 2016; Du et al., 2018; Li et al., 2019). Retigabine (RTG) was developed some 20 years ago as an anti-epileptic drug that acts by augmenting M current (Main et al., 2000; Rundfeldt and Netzer, 2000; Tatulian and Brown, 2003; Wuttke et al., 2005) and is widely used in research labs. Retigabine induces a hyperpolarizing shift of K_*v*_7.2-5 channel activation (but not K_*v*_7.1), resulting in current enhancement at potentials positive to −80 mV (Tatulian et al., 2001). However, although FDA-approved, its long-term side-effects (e.g., dilation of smooth muscle, blue tinting to skin over time) has led to its withdrawal from the market. Recently, however, a plethora of more selective “next-generation” M channel-targeting compounds have been developed. That may make manipulation of M current a modality used for myriad of brain disorders and insults, besides as anti-convulsants. In this review we will explore some of the possible new therapeutic uses of pharmacological M-current manipulation in treating brain dysfunction.

## M Current and Neurovascular Injuries

Two research groups first explored the role of M current during metabolic stress induced by oxygen and glucose deprivation (OGD) using cell culture models. They observed RTG-induced enhancement of M current to significantly reduce neuronal death in organotypic cultures of hippocampal slices subjected to 30 min of OGD, whereas M-current block with XE991 (Zaczek et al., 1998) resulted in increased neuronal death (Boscia et al., 2006; Gamper et al., 2006). Similar observations were reported by Barrese et al. (2015), using rat caudate brain slices. All these groups observed that OGD-induced damage was reduced by pharmacological M-current augmentation. Therefore, it seemed possible that pharmacological M-current enhancement could reduce brain damage after a stroke. Indeed, our group showed M-current augmentation to be neuroprotective after occlusive stroke. M-current augmentation strongly reduced stroke-induced neuronal death, the maladaptive immune response, and locomotor deficits (Bierbower et al., 2015). In a rat model, RTG treatment impaired stroke-induced increases in blood brain barrier (BBB) permeability, opening of tight junctions from microvascular endothelial cells, and cerebral infarct area (Zhao et al., 2018). An evident connection between stroke and the previous OGD models is that both involve cellular metabolic stress.

More recently, Vigil et al. (2020) also showed pharmacological M-current augmentation to prevent brain damage after traumatic brain injury (TBI). With only one *i.p.* injection of RTG 30 min post-injury, we observed significant reductions in post-traumatic seizures and seizure susceptibility, cellular energetic demand, the maladaptive inflammatory/immune response, breakdown of the BBB, and cell death (Vigil et al., 2020). Thus, we believe that prevention of initial TBI-induced hyperexcitability, even before a post-traumatic seizure can occur, severely hampered the damaging TBI-induced cascade of events. Interestingly, an increase in K_*v*_7.2 expression in TBI-subjected animals treated with RTG was observed in cortical and dentate gyrus hippocampal cells up to 6 days after TBI, although this transcriptional up-regulation is likely not to last much longer than 10 days (Carver et al., 2020; Vigil et al., 2020). As RTG has a half-life of 2 h in animals (Valeant Pharmaceuticals), it is reasonable to assume that M-current augmentation facilitated a later increase in K_*v*_7.2 transcription in neurons that survive the insult. This elevated expression of the *kcnq2* gene could represent a second longer-term therapeutic window, as one could take advantage of the increased expression of K_*v*_7.2 channels to maximize the effects of therapeutic treatment. Recently, acute RTG administration was also reported to improve pain and motor neuron recovery after spinal-cord injury (SCI). Wu et al. (2020) observed RTG treatment to be effective up to 3 days after SCI if local delivery of RTG was performed by a pump implant.

**FIGURE 1:**
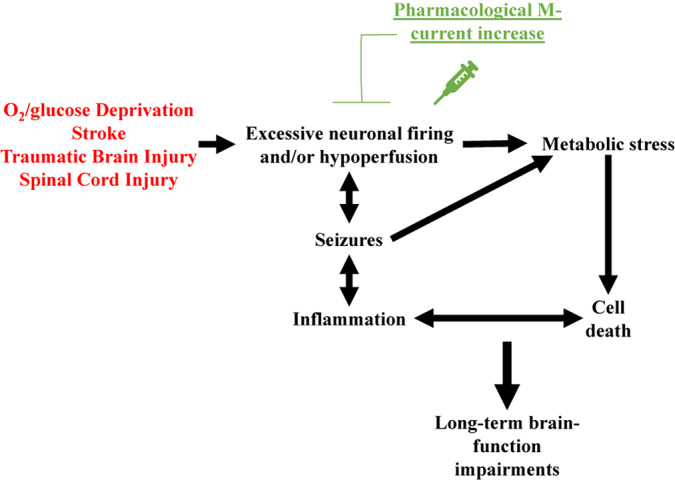
Schematic representation of the over-arching hypothesis proposed to explain the therapeutic effects of pharmacological M-current augmentation after O_2_/glucose deprivation, stroke, traumatic brain injury and spinal cord injury. These neurovascular insults all share brain hypoperfusion and/or excessive neuronal firing, leading to cellular metabolic stress, cell lysis. Cell death hyper-activates the immune system of the brain, increasing insult-related inflammation. If uncontrolled, this hyper-inflammation may result in further cell death, creating a deleterious cycle of positive feedback between these events. Insult-induced excessive neuronal firing also promotes seizures, which further increase cellular metabolic stress and enhanced inflammatory responses, facilitating the occurrence of more seizures. Acute pharmacological M-current increase post-insult stops this cascade of events by reducing the initial increased cellular energy demand.

Based on the above, a reasonable hypothesis is that pharmacological M-current augmentation reduces neuronal firing after TBI, SCI and, stroke, and as in the OGD model, reduces cellular energy demand. Therefore, reducing Na^+^/K^+^ ATPase activity, osmotic unbalance, and cell lysis. This hypothesis is summarized in Figure 1. However, we are only beginning to use *in vivo* models to confirm the cause of cell death after post-traumatic seizures. Moreover, the various elements of the injury-induced cascade of events are likely to further cross-activate each other resulting in the secondary injury often observed in TBI (Beez et al., 2017; Simon et al., 2017), SCI (Ahuja et al., 2017), and stroke (Hemphill et al., 2015; Beez et al., 2017). By initially preventing this cascade of events at the start, M-current augmentation should have long-lasting beneficial effects in these neurovascular injury events (Figure 1).

The secondary effects of TBI can be observed in epileptogenesis in which the injury converts a healthy brain into a brain in which synchronous neuronal activity and seizures are more likely to occur. Traumatic brain injury is responsible for 20% of symptomatic epilepsies and 5–6% of all epilepsy (Garga and Lowenstein, 2006). Higher risk of post-traumatic epilepsy may persist for up to a decade after an initial TBI, but an indeterminate latent period can last between months and years without any presentation of overt seizures (Frey, 2003; Christensen et al., 2009; Lowenstein, 2009). Hence, prior to seizure presentation, TBI must induce pathophysiological changes in the brain that increase seizure susceptibility and epileptogenesis. Post-traumatic epileptogenesis entails a wide scope of regulatory plasticity from many different ion channels, including GABA_*A*_ receptors, HCN channels, and K_*v*_7 channels, which often provide inhibitory opposition in response to neuronal hyperexcitability. However, both excitatory and inhibitory circuit reorganizations after TBI lead to maladaptive synaptic connectivity, contributing to epileptogenesis (Hunt et al., 2013). Due to the capacity of M-channel openers to act as an inhibitory force to the brain during susceptible periods of the post-traumatic cascade, they could provide control, and possibly prevention, of TBI-induced epilepsy.

Another role played by K_*v*_7 channels, specifically K_*v*_7.4 and K_*v*_7.5, are as regulators of excitability in blood vessels smooth muscle (Yeung et al., 2008; Joshi et al., 2009). Thus, it is possible that part of the beneficial effect of M-current augmentation might be ascribed to an acute increase in blood flow that would lead to a greater supply of glucose and O_2_ to support metabolic demands. However, the dilation of bladder smooth muscle, leading to urinary incontinence, has been suggested to be due to RTG actions on afferent nerve activity, rather than direct regulation of bladder myocyte contraction (Tykocki et al., 2019). In TBI and stroke models, increases in BBB permeability and infarct area were reduced by RTG treatment (Zhao et al., 2018; Vigil et al., 2020). Zhao et al. (2018) suggest that RTG may reduce BBB permeability by inhibition of injury-induced increase in expression of protein kinase C delta (PKCδ) and of the extracellular matrix proteinases, MMP-2 and MMP-9. Phosphorylation by PKCδ activates MMP-2/9, which degrades tight junction-associated proteins of cerebral vascular endothelial cells, resulting in increased BBB permeability. How RTG treatment reduces injury-induced expression of MMP-2/9 and PKCδ is unknown. Additionally, more experiments measuring brain blood flow and BBB permeability at different time points after injury in animals treated with RTG are still necessary to further investigate this matter.

An additional confound to consider is that RTG seems to affect other ion channels besides K_*v*_7 channels. Retigabine at 10 μM concentration reduces K_*v*_2.1 current by ∼20% and at 100 μM, RTG inhibits ∼80% of K_*v*_2.1 current (Stas et al., 2016). Retigabine at 10 μM also reduces current throughout L-type voltage-gated Ca^2+^ channels by >50% (Mani et al., 2013). Experimental evidence shows that RTG also acts on GABA_*A*_ receptors at concentrations above 10 μM (Treven et al., 2015). Inhibition of these channels may also play a role in the aforementioned therapeutic effects. For treatment of epilepsy in patients, the mean free average plasma concentrations of RTG is approximately 0.83 μM and maximum mean free plasma concentrations (Cmax) is approximately 1 μM (Gunthorpe et al., 2012). Hence, if the same doses are used for treatment of other diseases/injuries, off-target effects are likely to be avoided.

## M Current and Drug Addiction

### Alcohol Addiction

**FIGURE 2:**
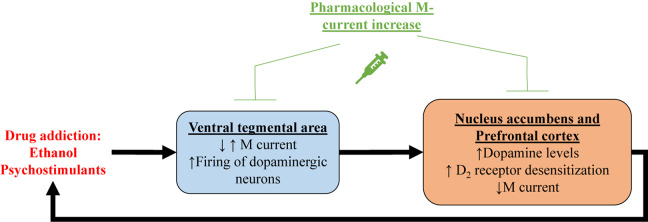
Shown is a proposed mechanism by which drug addiction may involve M current. Psychostimulant-induced increase in dopamine levels within the ventral tegmental area (VTA)could correlate with decreased M current in dopaminergic neurons, and/or increased M current in GABAergic neurons. In either case, increase firing of dopaminergic neurons in the ventral tegmental area releases more dopamine in the nucleus accumbens and the prefrontal cortex, inducing reward. Prolonged increases in dopamine levels result in desensitization of dopamine receptors. Concomitantly, M current is reduced in nucleus accumbens and prefrontal cortex, possibly caused by dopamine receptor desensitization. All of these addiction-induced alterations lead to increased drug intake, creating a deleterious cycle, which can be broken by pharmacological M-current increases in all of these brain regions.

One of the first demonstrations of the relationship between M current and drugs of abuse centers on alcohol addiction. Moore et al. (1990) showed that M current from hippocampal CA1 pyramidal neurons was inhibited by ethanol. In that same year, M current was recorded for the first time in ventral tegmental area (VTA) neurons (Lacey et al., 1990), a region that plays a key role in reward, mood and drug addiction (Di Chiara and Imperato, 1988; Lammel et al., 2012; Morales and Pickel, 2012), including to alcohol (Gessa et al., 1985; Rodd-Henricks et al., 2000). Koyama et al. (2007) showed that ethanol increases spontaneous firing frequency and suppresses M current in VTA dopaminergic neurons, with a correlation between the two actions. Ethanol seems to inhibit K_*v*_7.2/7.3 heteromers by a PIP_2_-related mechanism (Kim et al., 2019). Ethanol has also been shown to reduce K_*v*_7.2 trafficking to the membrane in neurons of the nucleus accumbens (NAc; McGuier et al., 2016), a region that is heavily innervated by the VTA and fundamental for reward and drug addiction (Weiss et al., 1993; Robinson and Berridge, 2003; Morales and Pickel, 2012). Retigabine injection, either systemically or into the NAc, significantly reduced voluntary ethanol consumption in rats, without any significant effect in sucrose or water consumption, whereas injection of XE991 increased it (Knapp et al., 2014; McGuier et al., 2016, 2018). Finally, systemic treatment with the K_*v*_7.2 and K_*v*_7.4 opener, ML213 (Yu et al., 2010), also reduced ethanol intake in rats (McGuier et al., 2018). Taken together, this evidence suggests regulation of M current to be linked to alcoholism, and that M-current augmentation may represent a mode of therapeutic intervention to treat alcoholism disease (Figure 2).

### Psychostimulant Addiction

Pharmacological M-current augmentation has also been tested as a treatment for addiction to the psychostimulants, cocaine, methylphenidate (Ritalin) and phencyclidine (PCP) in rat models (Hansen et al., 2007). Retigabine injection was shown to significantly reduce cocaine, methylphenidate and PCP-induced locomotor activity and c-Fos expression in the NAc and the primary motor cortex. Retigabine treatment also impaired methylphenidate-induced overflow of dopamine in the striatum (Hansen et al., 2007). More recently, Parrilla-Carrero et al. (2018) showed that training to self-administer cocaine reduced spike frequency adaptation (SFA) and AHPs in a subpopulation of prelimbic prefrontal cortex (PL-PFC) neurons. This increase in excitability was resistant to extinction training and enhanced by a cued reinstatement test. These neurons also show decreased M current amplitudes and reduced sensitivity to dopamine. *Ex vivo* treatment with RTG restored the SFA and AHPs of these PL-PFC neurons. Moreover, RTG injection directly into the PL-PFC was shown to reduce reinstatement-induced drug-seeking behavior (Parrilla-Carrero et al., 2018), a model of relapse in rodents. Psychostimulants are known to increase dopamine levels in the brain, resulting in desensitization of D_2_ receptors (D_2_Rs; Volkow et al., 2010; Juarez and Han, 2016). Experiments with heterologously expressed dopamine D_2_R and K_*v*_7.1-7.4 channels revealed D_2_R stimulation to increase M current in a mechanism involving G proteins of the G_*o/i*_ subtype (Ljungstrom et al., 2003). Hence, it is possible that the decrease of M-current amplitudes observed with psychostimulants is related to desensitization of D_2_Rs, involving diminished D_2_R facilitation of K_*v*_7 channel opening (Figure 2). This hypothesis could explain the hyperexcitability observed in VTA and PL-PFC. Reduction in membrane levels of K_*v*_7 channels could also play a role in psychostimulant-induced reduction of M current amplitudes.

In brief, addiction to both ethanol and psychostimulants seem to result in reduced M-current expression and/or amplitudes, and this reduction may be related to enhanced drug-seeking behavior (Figure 2). Indeed, pharmacological M-current augmentation has shown beneficial effects in a number of addiction models. We believe the VTA to be an ideal target for the use of pharmacological M-current augmentation as a novel treatment for addiction, although we do not yet know if the mechanism of action would be due to changes in the excitability of VTA dopaminergic neurons, GABAergic neurons, or both. Ventral tegmental area projections are the main source of dopamine in all the brain regions mentioned above (Ikemoto, 2007; Ferreira et al., 2008; Hosp et al., 2011; Morales and Pickel, 2012; Han et al., 2017). Even though RTG injection into the VTA does reduce ethanol consumption (McGuier et al., 2018), for example, intra-VTA injections are not of course feasible clinically. Nonetheless, the unique composition of K_*v*_7 channels in the VTA could represent a therapeutic opportunity. Ventral tegmental area expresses high levels of neuronal K_*v*_7.4 subunits (Li et al., 2017), compared to neurons from other regions in the brain, in which K_*v*_7.4 has little to no expression (Kharkovets et al., 2000; Saganich et al., 2001; Greene and Hoshi, 2017). This could allow for the use of drugs that specifically target K_*v*_7.4, such as fasudil (Li et al., 2017), as a treatment for addiction. However, continuous pharmacological M-current augmentation through K_*v*_7.4 channels is likely to induce hearing and blood pressure problems (Kharkovets et al., 2000; Kharkovets et al., 2006; Yeung et al., 2008; Joshi et al., 2009). Thus, more preclinical studies are necessary for pharmacological M-current augmentation to be used as a treatment for drug addiction.

## M Current and Mood Disorders

### Depression

**FIGURE 3:**
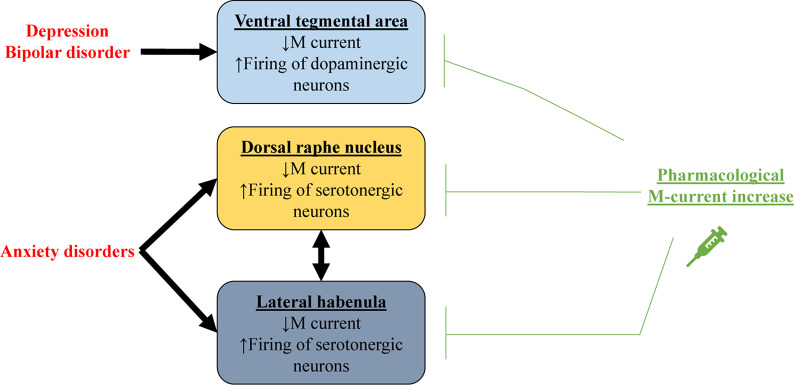
Schematic representation showing how decreased M current could be related to mood disorders. In depression and bipolar disorder, impaired M current in dopaminergic neurons of the ventral tegmental area (VTA) results in excessive firing. For anxiety disorders, M current is decreased in serotonergic neurons of the dorsal raphe nucleus and the lateral habenula. Pharmacological M-current increase in these regions could thus be used as a therapeutic tool for treating these mood disorders.

The VTA also plays a major role in mood disorders such as depression (Nestler and Carlezon, 2006; Figure 3), a widespread chronic illness characterized by low mood, lack of energy, sadness, and anhedonia (Cui, 2015). Pharmacological M-current augmentation, either systemically or in the VTA, reduces depression-like behavior in the social defeat depression mouse model, measured by different depression paradigms (Friedman et al., 2016). Additionally, intra-VTA viral vector transfection of K_*v*_7.3 channels also reduced depression like behavior, as did anterograde expression of K_*v*_7.3 in the NAc via intra-VTA injections of viral vector (Friedman et al., 2016). Li et al. (2017) observed that systemic pharmacological augmentation of K_*v*_7.4 channels with fasudil also reduced depression-like behavior and excitability of VTA neurons in the social defeat depression mice model.

#### Bipolar Disorder

Bipolar disorder is a severe chronic mood dysfunction that is characterized by oscillation between episodes of depression and of mania (American Psychiatric Association, 2013). During manic episodes, patients experience euphoria, hyperactivity, and high levels of risk-taking behavior (American Psychiatric Association, 2013). In rodents, manic episodes can be modeled by injection of amphetamine (AMPH), combined with the benzodiazepine, chlordiazepoxide (CDP). The combination of these drugs induces hyperactivity and this phenotype can be examined with drugs used for treating bipolar disorder in the clinic, such as lithium (Dencker et al., 2008; Redrobe and Nielsen, 2009). M-current augmentation has also displayed beneficial effects in rodent models of mania (Figure 3). Studies have found that administration of RTG 30 min before testing impaired AMPH + CDP-induced hyperactivity with no effects on basal locomotor activity (Dencker et al., 2008). Redrobe and Nielsen (2009) observed that enhancement of M current with ICA-27243, which selectively augments currents from K_*v*_7.2/7.3 channels, also reduced AMPH + CDP-induced hyperactivity. On the other hand, enhancement of K_*v*_7.4-7.5 currents by BMS-204352 did not have significant anti-manic effects. Further studies using the AMPH + CDP model showed RTG to reduce the AMPH + CDP-induced increase in cellular metabolic demand in the thalamus, striatum and retrosplenial cortex (Kristensen et al., 2012). Furthermore, intravenous injection of RTG was shown to reduce AMPH-induced locomotor activity, neuronal firing in the VTA, and dopamine release in the NAc, whereas XE991 had the opposite effect (Sotty et al., 2009). Retigabine was also shown to impair sensitization after multiple AMPH injections in rodents (Dencker and Husum, 2010).

A new genetic dimension involves glycogen synthase kinase 3 beta (GSK3β), which is best known for intra-nuclear re-phosphorylation of the transcription factor, nuclear factor of activated T-lymphocytes (NFAT), which despite its name, is ubiquitous in brain and critical to synaptic plasticity (Graef et al., 1999). Retigabine was found to increase phosphorylation of GSK3β in hippocampus and in the pre-frontal cortex (Kristensen et al., 2012). Such phosphorylation of GSK3β at serine 9 reduces kinase activity, similar to the effect of standard anti-manic agents, such as lithium (Stambolic et al., 1996; De Sarno et al., 2002). Using *in vitro* studies, GSK3β was seen to phosphorylate K_*v*_7.2 subunits, suppressing M current and lithium impairs this phosphorylation, rescuing M current (Borsotto et al., 2007). This relationship could offer a plausible novel target for the treatment of bipolar disorder.

Valproic acid (VPA) is an anti-epileptic agent commonly used in the clinic as a mood stabilizer for treatment of patients with bipolar disorder (Chiu et al., 2013; Cipriani et al., 2013). Using *in vitro* and *in vivo* mice models, part of the antiepileptic effect of VPA was shown to be due to inhibition of muscarinic-induced suppression of M current and to be dependent of K_*v*_7.2 phosphorylation at S558 (Kay et al., 2015; Greene et al., 2018). It is possible that the beneficial effect of VPA as a mood stabilizer may also be related to drug-induced M-current increases. Nonetheless, VPA has various mechanism of action that are not related to M current (Tomson et al., 2016; Collins-Yoder and Lowell, 2017). Therefore, a direct link between VPA-induced facilitation of M current and its effect as a mood stabilizer remains to be proven.

In humans, associations between bipolar disorder and single nucleotide polymorphisms (SNPs) in the *kcnq2* gene have been found (Borsotto et al., 2007; Judy et al., 2013). These SNPs disturb the interaction of K_*v*_7.2 with ankyrin G (Borsotto et al., 2007) and protein phosphatase 2A (Judy et al., 2013), which could impair channel assembly and dephosphorylation, respectively. Finally, bipolar patients have decrease methylation of exon 11 in the *kcnq3* gene, resulting in lower expression of K_*v*_7.3 (Kaminsky et al., 2015).

#### Anxiety

Both BMS-204352 and RTG have anxiolytic-like effects in the zero-maze and marble-burying rodent paradigms. Those effects were blocked by XE991 without any observed motor alterations, supporting pharmacological M-current enhancement as an anxiolytic treatment (Korsgaard et al., 2005). Anxiolytic-like dose-dependent effects of RTG were also observed in the conditioned emotional-response paradigm (Munro et al., 2007). As proposed by Hansen et al. (2008), the anxiolytic-like effects of BMS-204352, observed by Korsgaard et al. (2005), suggest K_*v*_7.4 and 7.5 to play an important role in anxiety. Immunostaining experiments show K_*v*_7.4 channels to be highly expressed in serotonergic neurons of the dorsal raphe nucleus (DRN; Hansen et al., 2008; Zhao et al., 2017), a region of the brain known to play a central role in anxiety regulation (Graeff et al., 1997; Lowry et al., 2008). For example, increased activity of serotonergic neurons from the DRN are observed in rodent models of induced anxiety (Maier and Watkins, 2005). The excitability of serotonergic neurons from the DRN can be manipulated by pharmacological and genetic manipulation of K_*v*_7.4 (Zhao et al., 2017). Hence, pharmacological M-current augmentation may have anxiolytic effects due to an increase in M current composed of K_*v*_7.4 homomers in serotonergic DRN neurons, resulting in reduced firing (Figure 3). Corroborating with this hypothesis, pharmacological M-current augmentation has been shown to reduce preoperative anxiety of human patients (Yadav et al., 2017). M current in the lateral habenula (LHb) also seems to play a role in anxiety disorders. Hyperexcitability in LHb was observed in a mouse model of ethanol withdrawal, concomitant with reduced M current, specifically from K_*v*_7.2 and 7.3. Additionally, infusion of RTG in the LHb impaired ethanol withdrawal-induced anxiety behavior (Kang et al., 2017). Curiously, intra-LHb injection of SB242084, an antagonist of the serotonin receptor 5-HT_2__*C*_, also reduced ethanol withdrawal-induced anxiety behavior and increased K_*v*_7.2 and 7.3 membrane protein levels (Fu et al., 2020). Serotonin 5-HT_2__*C*_ receptors are coupled to G_*q*__/__11_ and therefore activate phospholipase C (Martin et al., 2014), which could modulate M current. But how these events could affect K_*v*_7 channel membrane levels remains to be understood.

The evidence presented in this section highlight the promise of pharmacological M-current augmentation to be an effective treatment for multiple mood disorders, with different specificities of brain regions and channel subunits among the disorders. M-current regulation of dopaminergic VTA neurons may play a major role in depression and bipolar disorders (Figure 3). The VTA is an interesting therapeutic target due to its peculiarly high expression of K_*v*_7.4 compared to other brain regions (Kharkovets et al., 2000; Saganich et al., 2001; Greene and Hoshi, 2017). Nevertheless, brain-specific drug delivery would presently be necessary to avoid peripherical effects (Kharkovets et al., 2000; Kharkovets et al., 2006; Yeung et al., 2008; Joshi et al., 2009). High expression of K_*v*_7.4 channels can also be found in the DRN, where it is a potential therapeutic target for anxiety disorders, although for such disorders, augmentation of K_*v*_7.2 and 7.3 in the LHb may also be necessary/beneficial. It is important to remember that the LHb and the raphe nuclei have reciprocal innervations between each other (Vertes et al., 1999; Yang et al., 2008; Metzger et al., 2017; Zhang et al., 2018). Moreover, M current might even be important in the etiology of bipolar and anxiety disorders (Borsotto et al., 2007; Judy et al., 2013; Kaminsky et al., 2015; Kang et al., 2017; Fu et al., 2020). It is likely that in both disorders, regulation of M-current by serotonergic and dopaminergic receptors (Ljungstrom et al., 2003; Fu et al., 2020) is disturbed by disease-induced alterations in these neurotransmitters and their receptors.

## Conclusion

Currently pharmacological M-current manipulation is only approved by the FDA for treatment of epilepsy, although as mentioned above, RTG is off the market and its predecessor, flupertine (Szelenyi, 2013), has unacceptable liver toxicity (Puls et al., 2011; Michel et al., 2012). However, the plethora of “next-generation” M-channel openers (Miceli et al., 2018), of which many were highlighted at the recent International K_*v*_7 symposium in Naples, Italy in 2019, show great translational promise. Animal research indicates M current to be a therapeutic target for multiple brain disorders, including those with no current treatments, such as TBI and psychostimulant addiction. *But which compound should be tested first for these?* RTG has a clinical history as an adjunctive treatment for epilepsy (Brodie et al., 2010; Tompson et al., 2013). Thus, repurposing RTG could represent the fastest way to the clinic, as it is already FDA-approved (in oral, but not injectable, form) and is the compound most incorporated into preclinical research involving M-channel augmentation. However, the prolonged use of RTG entails adverse side effects, such as reversible skin discoloration, retinal pigmentation abnormalities, cognitive changes, and urinary incontinence (Beacher et al., 2015; Zaugg et al., 2017). The discoloration effects are largely due to the properties of the dimerized compound that may be mitigated in more recent derivatives of RTG that have yet to undergo clinical trials. It has been suggested that other side effects may also be reduced by identifying RTG-derivatives that are more potent and selective for K_*v*_7.2/7.3 channels (Grunnet et al., 2014; Kumar et al., 2016). Another benefit of selectivity for K_*v*_7.2/7.3 has been suggested in the treatment of tinnitus involving M-current dependent plasticity in the dorsal cochlear nucleus (Li et al., 2013; Kalappa et al., 2015). As previously mentioned, RTG also has dose-dependent side effects on K_*v*_2.1 (Stas et al., 2016), L-type voltage-gated Ca^2+^ channels (Mani et al., 2013), and GABA_*A*_ receptors (Treven et al., 2015). In addition, newer insights suggest that GABA interacts with, and activates, certain K_*v*_7 subtypes due to a conserved binding pocket (Manville and Abbott, 2018, 2020). Thus, the use of more potent and specific M-channel compounds, as well as alternative strategies to regulate M-channel transcription, need to be further explored.

Much is still unknown about how M current is involved in the variety of neurological diseases mentioned in this review. Even basic preclinical tests remain to be performed for most of them. For example, for the new RTG-derivatives the minimum and maximum effective doses and possible side effects of long-term use are unknown. In neurovascular injuries, the necessity of one or only a few doses of treatment after the injury should eliminate the risks associated with long-term use of a drug. Since mood disorders are chronic diseases, the clinical use of pharmacological M-current augmentation necessitates prolonged use, presenting an extra challenge. It is also necessary to start investigating brain-specific delivery of M-current modulators. Addiction is a case that remains a bigger challenge. In rodent models, M current has been described to reduce both drug intake and relapse-like drug seeking behavior. Translating this to the clinic, M-current augmentation might be useful for treatment of an active drug user and/or to reduce relapse of a patient that is no longer using the drug. Both possibilities can only be truly tested in clinical trials. Pharmacological M-current augmentation is likely to be a therapeutic tool for a spectrum of pathological situations, as discussed here. However, there is still a long road ahead until clinical trials establish the true value of this mode of therapeutic intervention.

## Author Contributions

All authors wrote the manuscript.

## Conflict of Interest

The authors have submitted a pending US patent for the use of K_*v*_7 channel openers as a novel therapy for traumatic brain injury, held in the name of Advanced Neuroresearch Therapeutics, LLC.
